# Initiation and cessation of mental healthcare after mental health screening in primary care: a prospective cohort study

**DOI:** 10.1186/s12875-018-0864-9

**Published:** 2018-11-17

**Authors:** Christine Geyti, Else-Marie Dalsgaard, Annelli Sandbæk, Helle Terkildsen Maindal, Kaj Sparle Christensen

**Affiliations:** 1 Section for General Medical Practice, Department of Public Health, Aarhus University, Bartholins Allé 2, 8000 Aarhus, Denmark; 2 Research Unit for General Practice, Aarhus, Bartholins Allé 2, 8000 Aarhus, Denmark; 3 Section for Health Promotion and Health Services, Department of Public Health, Aarhus University, Bartholins Allé 2, 8000 Aarhus, Denmark

**Keywords:** Mental health, Mental health screening, Mental healthcare, General practice, Primary healthcare, Health promotion, Preventive health services

## Abstract

**Background:**

Mental health (MH) problems have considerable personal and societal implications. Systematic MH screening may raise general practitioners’ (GP) awareness of the current need of treatment in their listed patients. The focus of MH screening has so far been on increasing diagnostic rates and treatment of mental disorders, whereas cessation of MH treatment after normal test results has rarely been studied. This study aims to examine the mental healthcare trajectories after MH screening combined with feedback on both positive and negative screening results to the GP.

**Methods:**

This prospective cohort study is based on data from 11,714 randomly selected individuals aged 30–49 years, who were invited to a preventive health check in Denmark during 2012–2015. A total of 5970 (51%) were included. MH status was assessed using the SF-12 Health Survey Mental Component Summary score, and scores were categorised into poor, moderate, and good MH. ‘Mental healthcare’ within 1 year of follow-up covered the following MH support: psychometric testing by GP, talk therapy by GP, contact to psychologist, contact to psychiatrist, and psychotropic medication.

**Results:**

MH was found to be poor in 9%, moderate in 25%, and good in 66% of participants. After 1 year, mental healthcare was initiated in 29% of the participants with poor MH who did not receive mental healthcare at baseline, and mental healthcare was ceased in 44% of the participants with good MH who received mental healthcare at baseline. Odds ratio (OR) for initiation of mental healthcare was associated with worse MH screening status: poor MH: OR 7.1 (5.4–9.4), moderate MH: OR 2.4 (1.9–3.1), compared to those with good MH. OR for cessation of mental healthcare was associated with better MH screening status: good MH: OR 1.6 (1.1–2.6), moderate MH: OR 1.6 (1.0–2.4), compared to those with poor MH. Initiation and cessation of mental healthcare appeared to be time-related to the MH screening.

**Conclusions:**

MH screening combined with feedback on both positive and negative screening results to the GP may contribute to relevant initiation and cessation of mental healthcare.

**Trial registration:**

Registration of the Check Your Health-trial: ClinicalTrials.gov (NCT02028195), 7 March 2014.

**Electronic supplementary material:**

The online version of this article (10.1186/s12875-018-0864-9) contains supplementary material, which is available to authorized users.

## Background

Mental health (MH) problems have considerable personal and social implications due to the negative impact on quality of life, health, productivity, and economic costs [1]. The World Health Organization (WHO) defines MH as ‘a state of well-being in which every individual realizes his or her own potential, can cope with the normal stresses of life, can work productively and fruitfully, and is able to make a contribution to her or his community’ [2]. In addition, WHO predicts that mental illness will be the leading cause of burden of disease by 2030 [3].

In Denmark, GPs act as gatekeepers to secondary care, and the majority of MH problems are handled by the general practitioner (GP) [4]. However, the accuracy of MH diagnoses made by GPs has been questioned [5]. The gold standard for identifying mental disorders in a psychiatric setting is a structured diagnostic interview [6], but time pressure, lack of remuneration, and lack of training makes this approach difficult to implement in general practice.

Previous studies have explored screening for sub-threshold MH problems and diagnosable mental disorders as a tool to support the diagnosis and management of patients with MH problems in general practice, e.g. the PsyScan study by Gidding et al. [7]. The PsyScan trial evaluated the effect of using a screening tool for psychological problems and was aimed at primary care patients suspected by the GP to have psychological problems. The web-based screening tool provided feedback to the GP on the results. However, the GP received screening information only for individuals who were already suspected to have MH problems. Thus, the GPs were not made aware of new possible cases. In addition, the PsyScan study was limited by loss to follow-up, as is often the case in research focusing on MH.

Systematic MH screening has the potential to raise the GP’s awareness of individuals in need of initiation or cessation of treatment. Despite this dual potential, the primary focus of MH screening has been on increasing diagnostic rates and treatment of MH disorders [8]. The effect on discontinuation of MH treatment after normal test results has rarely been studied [9].

We conducted a study based on MH screening data collected as part of a large-scale municipality-wide population-based prevention programme entitled ‘*Check Your Health’* and provided feedback on the results to the GP [10]. The *Check Your Health* programme was launched in a mid-sized municipality in Denmark in 2012 in collaboration with the local GPs and was funded by the municipality. The overall aim of the programme was to improve the health outcomes of individuals aged 30–49 years living in the municipality. We used the Danish national registers to follow up on the MH trajectories, which implied that we had full follow-up information on both initiation and cessation of mental healthcare for all included individuals.

## Methods

### Aim

The overall aim of the study was to examine the mental healthcare trajectories after MH screening combined with feedback of both positive and negative screening results to the GPs.

We incorporated a general MH screening in a Danish population-based health prevention programme and transferred the results to the GP. We hypothesised that feedback on the patient’s MH status to the GP would be clearly associated with both initiation and cessation of mental healthcare.

### Study design

The Danish *Check Your Health* preventive programme [10] included a population-based preventive health check in a municipality followed by a face-to-face health check consultation with the participant’s GP and targeted behavioural programmes in the local health centre. The health check included MH screening in addition to risk assessment for long-term conditions, e.g. cardiovascular disease (CVD) and diabetes. All participants completed a web-based questionnaire on self-rated general health, mental health, and health behaviour. The results from the questionnaire and the clinical examination were printed immediately after the examination. The health professionals went through the results with the participants, and participants with a ‘risk’ profile (poor MH or any other pre-defined health risk measure [10]) were recommended to book a health check consultation with their GP. Health check results, including results on MH status, were incorporated into the GP’s electronic health record on the patient. The health check consultation with the GP was based on a shared agenda between the GP and the participant. National clinical guidelines governed how the patients were treated by the GP [10]. Additionally, after receiving the screening results, the GP could refer eligible individuals to focused behavioural programmes at the local health centre. These programmes specifically targeted participants in the *Check Your Health* programme [10]. An a priori calculation estimated that each GP would have one follow-up consultation every two to three weeks.

**Fig. 1:**
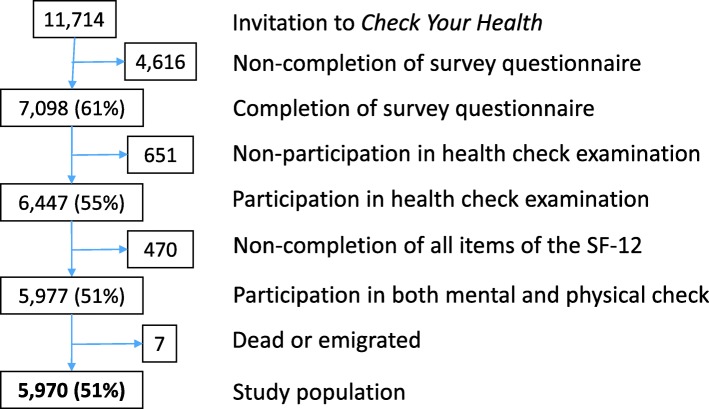
Flowchart of inclusion. Participants aged 30–49 years from Randers Municipality, Denmark, recruited from the *Check Your Health* preventive programme, 2012–2015

All 30- to 49-year-old citizens who lived in Randers Municipality in Denmark on 1 January 2012 (*n* = 26,216) were randomised to receive an invitation to the *Check Your Health* preventive programme in one of the following 5 years. This age range was the aim of the programme because of the potential to prevent development of both mental and physical disorders and possible complications [11]. We excluded citizens who had died, had moved from the municipality, or had terminal illness before the invitation. To comply with the capacity to perform the health checks in the health centre and the capacity of the GPs to follow up, the invitations were sent out on a weekly basis from 18 April 2012. As shown in Fig. 1, the present prospective cohort study was based on individuals invited during the first 3 years, i.e. 18 April 2012–1 April 2015 (*n* = 11,714). We included participants in the health check examination who completed the MH screening before 1 July 2015. We followed the cohort for 1 year after each participant’s date of MH screening. A small number of participants died or emigrated from Denmark before the end of the follow-up period (*n* = 7). In total, 5970 citizens formed the cohort. Information from the *Check Your Health* preventive programme on MH status, sex and age at randomization was linked to data from Danish registers through each participant’s unique personal identification number, which is assigned to all individuals with permanent residence in Denmark [12]. Information on cohabitation (married or living with a partner vs. living alone) and highest educational level was obtained from administrative registers managed by Statistics Denmark [12, 13]. Education was categorised into 0–10, 11–15, and > 15 years of education according to the International Standard Classification of Education by the United Nations’ Educational, Scientific and Cultural Organization (UNESCO) [14].

### Mental health screening

MH was assessed with the 12-item Short Form Health Survey (SF-12), version 2, Mental Component Summary (MCS) score [15]. Calculation of MCS scores, which were based on the 1998 SF-12 U.S. population norms, was performed for complete SF-12 surveys only [16]. Each of the 12 items of the SF-12 leads to a number of indicator variables. For example, the item assessing how much of the time you did work or activities less carefully than usual as a result of any emotional problems (such as feeling depressed or anxious) has five response choice categories: All of the time, most of the time, some of the time, a little of the time or none of the time. Each indicator variable is multiplied by its respective mental regression weight, and the products are summed to compute the MCS [16]. Thus, all items of the SF-12 are included in the MCS algorithm, and the MCS score encompasses general self-rated health, mood and anxiety symptoms, physical health, and functional limitations during the past 4 weeks [15]. Although the MCS does not target any specific psychiatric diagnoses, it has been validated against diagnoses of mental disorders [17–19]. MCS scores ranged from 0 to 100 on a continuous scale; higher score reflected better MH. We defined ‘poor’ MH as an MCS score of ≤35.76, ‘good’ MH as an MCS score of ≥48.26, and ‘moderate’ MH as an MCS score in between. The cut-off points were based on a Danish national health survey; ‘poor’ MH corresponded to the 10% of the Danish adult population with the lowest MCS scores, and ‘good’ MH corresponded to the 65% with the highest MCS scores [20].

### National health services

Approximately 98% of the Danish citizens are listed with a specific general practice [21]. Consultations and mental healthcare services from the GP are covered by the publicly funded Danish National Health Service (NHS). The content of the consultation (other than the recorded services provided) is not available from national registers. Referral from the GP is needed to obtain psychiatric specialist care (private psychiatrists bound by a collective agreement with the NHS or psychiatric hospitals including outpatient clinics), which is also free of charge for the patient. Patients who fulfil the referral criteria for psychological therapy pay only 40% of the psychologist fee, and the NHS covers the rest.

### Definitions of mental healthcare

We defined ‘mental healthcare’ as at least one of the following types of MH support recorded in the Danish national health registers: psychometric testing by GP, talk therapy by GP, contact to psychologist, contact to psychiatrist, or psychotropic medications (Table 1). These will hereafter be referred to collectively as ‘mental healthcare’.

**Table 1:** Definitions and sources of mental healthcare

Type of mental health follow-up support	Register	Notes
• Psychometric testing by GP	NHSR	Approved psychometric tests, e.g. diagnostic tests for depression or anxiety
• Talk therapy by GP	NHSR	By GPs receiving psychological supervision
• Psychologist	NHSR	After referral from GP
• Psychotropic medications	DNPR	Redeemed prescription of the following medication (ACT codes): antipsychotics (N05A), anxiolytics (N05B), hypnotics and sedatives (N05C), antidepressants (N06A), psychostimulant medication (N06B), anti-dementia drugs (N06D)
• Psychiatrist	NHSR/NPR	Private psychiatrists/psychiatric hospitals (inpatients and outpatients)

*ACT* Anatomical Therapeutic Chemical Classification, *DNPR* Danish National Prescription Register [38], *GP* general practitioner, *NHSR* (Danish) National Health Service Register [39], *NPR* (Danish) National Patient Register [40]

We defined ‘mental healthcare at baseline’ as at least one type of mental healthcare recorded within 3 months before the MH screening. We defined ‘initiation of mental healthcare’ as a recorded mental healthcare intervention preceded by no mental healthcare at baseline.

We defined ‘cessation of mental healthcare’ as no recorded MH support for 6 months among those who had received at least one type of the recorded MH support at baseline.

### Statistical analyses

Cumulative proportions of participants who initiated mental healthcare in the follow-up period were compared between participants with poor, moderate and good MH who did not receive mental healthcare at baseline. Cumulative proportions of participants with good and moderate MH who discontinued their mental healthcare in the follow-up period were compared with participants with poor MH who received mental healthcare at baseline. Proportions were compared using two-sample test of proportions. *P*-values of < 0.05 were considered statistically significant.

Univariate and multivariate logistic regression analyses were used for estimating crude and adjusted odds ratios (OR) for initiation and cessation of mental healthcare. In the multivariate analyses, we adjusted for covariates based on the four most frequent examined variables in research on healthcare use [22]; sex, age (continuous), highest educational level, and cohabitation status.

All estimates were reported with 95% confidence intervals (CI). Results with less than five observations were reported as < 5 (in accordance with the data protection regulations from Statistics Denmark [23]). Time intervals were one quarter of a year (91 days), except in the case of < 5 observations per quarter where the observations were transferred to the following quarter. The statistical analyses were performed on the remote server of Statistics Denmark. Stata software, version 14.2 (StataCorp, College Station, Texas), was used for all calculations. Missing data was not imputed.

## Results

A total of 51% (*n* = 5970) of the invited population participated in both the health check and the MH screening; 99.9% were followed for 1 year (Fig. 1). Men composed 49% of the study population (Table 2). The mean age was 42.3 years (42.2–42.5). One third had high level of education, half of participants had medium level of education, and the remaining participants had low level of education. One in five lived alone (Table 2). Compared to the study population, individuals who were excluded due to non-completion of all items of the SF-12 (*n* = 470) were significantly more likely to be characterised by disadvantaged socio-demographic status (Additional file 1: Table S1).

**Table 2:** Socio-demographic characteristics of the study population

	*n*	% (95% CI)
Participants	5970	
Sex		
Women	3018	50.6 (49.3–51.8)
Men	2952	49.4 (48.2–50.7)
Age, mean (95% CI)	42.3	(42.2–42.5)
Cohabitional status		
Living alone	1297	21.7 (20.7–22.8)
Cohabiting	4669	78.3 (77.2–79.3
Education (years)		
0–10	853	14.5 (13.6–15.4)
11–14	3022	51.2 (50.0–52.5)
> 15	2023	34.3 (33.1–35.5)

Participants in mental and physical health check, aged 30–49 years, from Randers Municipality, Denmark, recruited from the *Check Your Health *preventive programme, 2012–2015

A total of 9% of the study population had poor MH according to the MH screening, 25% had moderate MH, and 66% had good MH. One in three with poor MH (*n* = 168) had a health check consultation with their GP within 1 year after the health check, whereas only one in four with moderate MH (*n* = 364) and one in five with good MH (*n* = 848) had such consultation (Table 3).

**Table 3:** Mental healthcare within 1 year of follow-up

Follow-up support within one year after the health check	Poor MH		Moderate MH		Good MH	
	*n*	% (95% CI)	*n*	% (95% CI)	*n*	% (95% CI)
	545	9.1 (8.4–9.9)	1478	24.8 (23.7–25.9)	3947	66.1 (64.9–67.3)
Health check consultation^a^ with GP	168	30.8 (27.0–34.9)	364	24.6 (22.4–26.9)	848	21.5 (20.2–22.8)
Any mental healthcare	273	50.1 (45.8–54.4)	334	22.6 (20.5–24.8)	333	8.4 (7.6–9.3)
Psychometric testing, GP	80	14.7 (11.8–17.9)	74	5.0 (4.0–6.2)	73	1.8 (1.4–2.3)
Talk therapy, GP	66	12.1 (9.5–15.1)	64	4.3 (3.4–5.5)	58	1.5 (1.1–1.9)
Psychologist	43	7.9 (5.8–10.5)	34	2.3 (1.6–3.2)	36	0.9 (0.6–1.3)
Psychiatrist	79	14.5 (11.6–17.7)	52	3.5 (2.6–4.6)	27	0.7 (0.4–1.0)
Psychotropic prescription	206	37.8 (33.7–42.0)	249	16.8 (14.9–18.8)	252	6.4 (5.7–7.2)

Descriptive analyses are stratified on mental health screening status at the health check at *Check Your Health,* 2012–2015 (N = 5970)

Poor MH: MCS ≤ 35.76. Moderate MH: MCS > 35.76 to < 48.26. Good MH: MCS ≥ 48.26. GP: general practitioner. MCS: mental component summary (score from SF-12, v. 2, US norms of 1998). MH: Mental health. SF-12: 12-item Short Form Health Survey

^a^Follow-up consultation with GP prompted by results from the health check

Half of participants with poor MH, 23% of participants with moderate MH, and 8% of participants with good MH received mental healthcare during 1 year after the health check. Three in four participants who received mental healthcare had redeemed a prescription of psychotropic medication within 1 year after the health check (Table 3).

### Initiation of mental healthcare and contacts to GP

The analyses of initiation of mental healthcare included exclusively participants who did not receive mental healthcare at baseline. Mental healthcare was initiated among one in four participants with poor MH, one in eight participants with moderate MH, and one in 20 participants with good MH (Table 4).

**Table 4:** Initiation and cessation of mental healthcare within 1 year of follow-up

	Poor MH		Moderate MH		Good MH	
	n	% (95% CI)	n	% (95% CI)	n	% (95% CI)
	545	9.1 (8.4–9.9)	1478	24.8 (23.7–25.9)	3947	66.1 (64.9–67.3)
Mental healthcare at baseline						
Yes	179	32.8 (28.9–37.0)	218	14.7 (13.0–16.7)	171	4.3 (3.7–5.0)
No	366	67.2 (63.0–71.1)	1260	85.3 (83.3–87.0)	3776	95.7 (95.0–96.3)
Initiation and cessation in follow-up period						
Initiation of any mental healthcare^a^	106	29.0 (24.4–33.9)	146	11.6 (9.9–13.5)	193	5.1 (4.4–5.9)
Cessation of mental healthcare^b^	62	34.6 (27.7–42.1)	103	47.2 (40.5–54.1)	83	48.5 (40.1–56.3)

Descriptive analyses are stratified on mental health screening status at the health check at *Check Your Health,* 2012–2015 (*N* = 5970)

Poor MH: MCS ≤ 35.76. Moderate MH: MCS > 35.76 to < 48.26. Good MH: MCS ≥ 48.26. MCS: mental component summary (score from SF-12, v. 2, US norms of 1998). MH: Mental health. SF-12: 12-item Short Form Health Survey. Mental healthcare: psychometric testing by GP, talk therapy by GP, contact to psychologist, contact to psychiatrist, or psychotropic medication recorded in the Danish national health registers

^a^Among participants who did not receive mental healthcare at baseline

^b^Among participants who did receive mental healthcare at baseline

The feedback on MH status to the GP was associated with initiation of mental healthcare in a dose-response manner, and did not change significantly after adjustment for sex, age, educational level and cohabitation (Table 5).

**Table 5:** Odds ratios (OR) for initiation of mental healthcare within 1 year of follow-up (*n* = 5402)

Screening result	Crude OR (95% CI)	Adj.^a^ OR (95% CI)
Poor mental health	7.6 (5.8–9.9)	7.1 (5.4–9.4)
Moderate mental health	2.4 (1.9–3.0)	2.4 (1.9–3.1)
Good mental health	Reference	Reference

^a^Adjusted for sex, age, educational level, and cohabitation

**Fig. 2:**
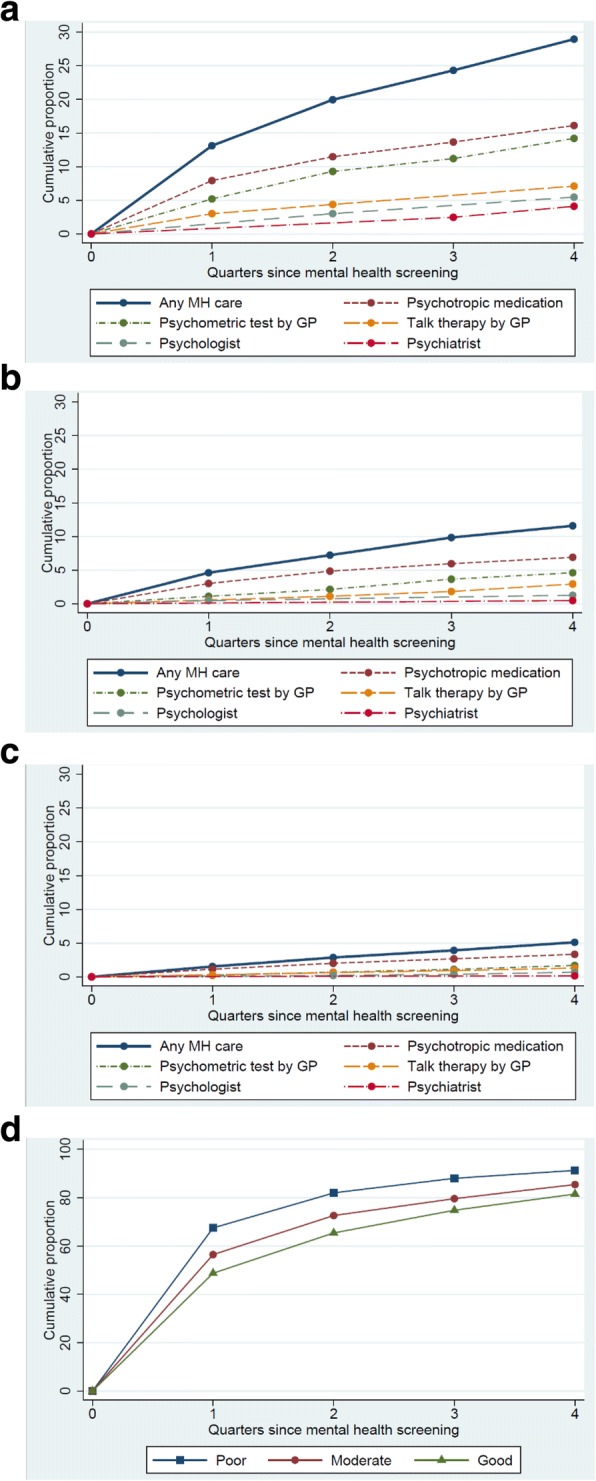
Initiation of mental healthcare and first contact to GP within 1 year of follow-up. Among participants who did not receive mental healthcare at baseline. *Initiation of mental healthcare*: **a**) poor MH (*n* = 366), **b**) moderate MH (*n* = 1260), **c**) good MH (*n* = 3776). *First contact to GP*: **d**) poor MH (*n* = 366), moderate MH (*n*= 1260), good MH (*n*= 3776)

A statistically significant larger proportion of new receivers of mental healthcare was seen within the first quarter compared to each of the following quarters among participants with poor or moderate MH (Fig. 2a and b).

Among participants with good MH, no difference was seen in the proportion of new mental healthcare receivers in the first quarter compared to each of the following quarters (Fig. 2c).

Among participants with poor MH who had not received mental healthcare at baseline, a gradual increase was seen in the proportion who had a psychometric test performed in general practice within the follow-up period (Fig. 2a).

Likewise, both the proportion who received talk therapy from the GP and the proportion who received psychotropic medication gradually increased. Less than five participants with poor MH and no mental healthcare at baseline consulted a psychologist within the first quarter after the MH screening. None of the participants had visited a psychiatrist within the first two quarters. Two thirds of those with poor MH and half of those with good MH visited the GP (*p* < 0.0001) within the first quarter (Fig. 2d). Within 1 year after the MH screening, 91% of those with poor MH and 81% of those with good MH had visited their GP for any reason (*p* < 0.0001) (Fig. 2d).

### Cessation of mental healthcare

**Fig. 3:**
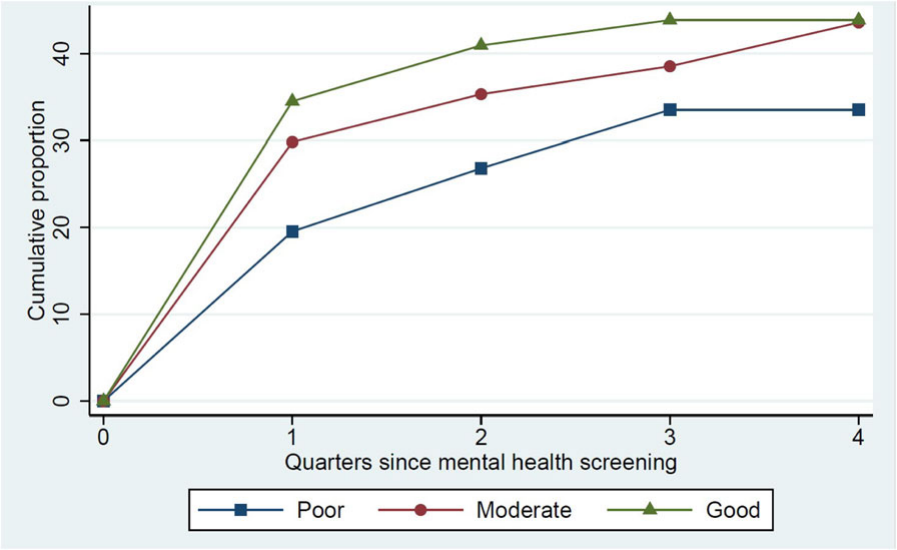
Cessation of mental healthcare within one year of follow-up. Among participants who received mental healthcare at baseline. Good MH (*n* = 171), moderate MH (*n* = 218), poor MH (*n* = 179)

The analyses of mental healthcare cessation included exclusively participants who received mental healthcare at baseline. The mental healthcare was ceased within the first 3 months after the MH screening for one in three participants with good MH (Fig. 3). This proportion was significantly larger than among participants with poor MH (20%). After 1 year, the mental healthcare was ceased for 44% of participants with good MH and 34% of participants with poor MH (*p* < 0.05) (Table 4). The feedback to the GP on MH status was associated with cessation of mental healthcare among participants with good MH and participants with moderate MH compared to participants with poor MH (*p* < 0.05) (Table 6). The results did not change after adjustment for sex, age, educational level and cohabitation.

**Table 6:** Odds ratios (OR) for cessation of mental healthcare within one-year of follow-up (*n* = 568)

Screening result	Crude OR (95% CI)	Adj.^a^ OR (95% CI)
Poor mental health	Reference	Reference
Moderate mental health	1.5 (1.0–2.3)	1.6 (1.0–2.4)
Good mental health	1.5 (1.0–2.4)	1.6 (1.1–2.6)

^a^Adjusted for sex, age, educational level, and cohabitation

## Discussion

### Main findings

This study is unique as it, to the best of our knowledge, is the first to examine the association between MH screening and both subsequent initiation or cessation of mental healthcare. We have described the major mental healthcare trajectories after a population-based MH screening with feedback to the GP on individual screening results. Reporting of poor MH status to the GP was associated with initiation of mental healthcare, whereas reporting of good MH status was associated with cessation of existing mental healthcare.

### Follow-up support after health check

Only one in three participants detected with poor MH at the health check had a follow-up health check consultation with the GP, however, nearly half of participants with poor MH received some kind of MH support from the public healthcare system. This difference may have two explanations. First, it is possible that the number of follow-up health check consultation with the GP was underestimated. If the participant made an appointment for a normal consultation rather than a *Check Your Health*-consultation, the GP may not have recorded the consultation as a follow-up health check consultation. Second, the GP may be aware of the participant’s MH problems, independently of receiving feedback on the MH screening result from the health check.

### Initiation of mental healthcare

The initiation of mental healthcare that took place within the follow-up period, was mainly performed within the first 6 months after the MH screening, and the mental healthcare was primarily provided by the GP. Contacts to psychologists and psychiatrists were mainly taken 6–12 months after the MH screening. Possible explanations could be that GPs seek to handle the patient’s MH problems first. If the MH problem does not improve, the GP may refer to secondary mental healthcare. Moreover, waiting time for appointments with specialist mental healthcare providers may contribute to the observed results [24]. The time relation indicates that the mental healthcare provided is associated with the MH screening and the feedback to the GP of the individual screening results. However, for three in four participants with poor MH who did not receive mental healthcare at baseline, no initiation of any mental healthcare was seen during the one-year follow-up. This result was surprising because most of them had visited their GP (for any reason) within the same period. As we had no information on the content of the consultations, we cannot know how many actually did have some kind of (unregistered) psychological support by the GP. Explanations for the low uptake of mental healthcare may be the stigma associated with MH problems [25] or the individual’s wish to handle the problems by themselves [26]. Another explanation for the low proportion of initiated mental healthcare could be that GPs have too few resources to handle complex health issues, such as MH problems, which often coexist with physical diseases [27]. As only 10–15 min are available per consultation, the GP may rather address the physical problems [28]. Furthermore, the low rate of contacts to psychologists may be due to out-of-pocket payment requirements for psychological appointments in Denmark. Removing the financial barrier may be a means to improve the access to and benefit from psychological treatment, as seen in the Improved Access to Psychological Treatment (IAPT) initiative in the UK National Health Service [29].

A recent Dutch study by Gidding et al., the PsyScan study, found somewhat similar results on several other parameters [7]. They used a different screening tool than the one used in the *Check Your Health* programme, but they found the same proportion of participants with positive screening results who received psychotropic medication (37%) at one-year of follow-up as we did (38%). Likewise, Gidding et al. identified a similar proportion of referrals to psychiatrists (12%) as we did in our study (15%). However, they found a much higher proportion of referrals to psychologists (17% vs. 8% in our study). An explanation for the observed difference could be that there is a substantial user fee for psychologist consultations in Denmark, whereas such consultations are largely free of charge in the Netherlands [30]. Gidding et al. further compared healthcare trajectories with a control group, who was identified only on the basis of the GP’s suspicion of psychological problems. They found that the screening-detected individuals had statistically insignificant higher odds of getting a referral to a psychologist or psychiatrist, but no increased odds of being prescribed psychotropic medication. However, the authors did not succeed in recruiting the calculated number of participants, and their study may therefore be under-powered to demonstrate an effect.

A meta-analysis of studies comparing feedback on depression screening results to the GP with routine care found an increased rate of recognition and a tendency towards higher rates of intervention for depression [8]. Thus, previous studies have found positive results on initiation of mental healthcare after screening, but the estimates are statistically insignificant. The studies included in the meta-analysis were markedly heterogeneous, and the authors are cautious not to draw any firm conclusions. Other studies suggest that systematic pre-screening of MH has the potential to increase the GP’s awareness of individuals in need of treatment [31].

### Cessation of mental healthcare

For nearly half of the participants who received mental healthcare at baseline and were rated to have good MH, the existing mental healthcare ceased within the follow-up period in the present study. The largest proportion ceased within the first 3 months after the MH screening, and only a marginal proportion ceased after 6 months. This could indicate that the GPs responded to the feedback on the participant’s good MH status. Possible reasons for continuing mental healthcare among the other half of participants with good MH are manifold: the GP may not have noticed the feedback in the participant’s electronic health record, or the participant may still need mental healthcare (for example patients with chronic or recurrent episodes of mental disorders for whom long-term use is advised) [32]. GPs should regularly re-evaluate the patients who receive psychotropic medication [32]. A post-hoc subgroup analysis revealed that among those with good MH who were prescribed psychotropic medication at baseline, the GPs had seen nearly all (94% (95% CI: 89–98)) at least once within the follow-up period. Thus, the GPs may have seen the screening results. However, certain reservations should be made as we had no access to information on the content of consultations. Thus, we do not know if MH was the subject of the consultation.

Previous studies have indicated that overtreatment is mainly explained by prolonged treatment rather than non-optimal treatment [33]. In a Dutch study, Eveleigh et al. assessed the effectiveness of a tailored recommendation to cease antidepressant medication [9]. They found that half of patients complied with the advice to stop their antidepressant treatment. However, only 6% succeeded, defined as ‘no antidepressant use during the preceding 6 months and the absence of a depressive or anxiety disorder during the one year follow up’ [9]. There are different possible explanations for the lower success rates in the Dutch study compared to ours. Eveleigh et al. focused solely on cessation of antidepressant medication among over-treated long-term antidepressant users, whereas we concentrated on any mental healthcare among participants with good MH screening results. Hence, short-term users of psychotropic medication formed part of our population. Cessation of psychotropic medication among long-term users is a well-known challenge [34], which may explain the low success rate in the Dutch study. In our study, some short-term users may have stopped their medical treatment due to side effects or lack of effect, which may explain part of the cessation in our study.

### Strengths and limitations

A major strength of the present large-scale population-based study is that it was implemented in the existing healthcare system and thus reflects realistic healthcare trajectories in Denmark and in other countries with a similar healthcare system and similar commissioning structures. We used national registers as a source of information on mental healthcare. Thus, we achieved virtually complete follow-up on nearly all participants (99.9%). Furthermore, registers are considered a better source to inform on utilisation of MH services than surveys [35]. However, we focused only on individuals aged 30–49 years, thus the results may not be valid for other age ranges.

To the best of our knowledge, our study is the first to evaluate the association between MCS score from the SF-12 and subsequent mental healthcare trajectories. The MCS score is a commonly used measure of generic MH in epidemiological research [15]. Moreover, it has been suggested as a useful screening tool for common mental disorders in the general population [17]. The MCS has been validated against depressive and anxiety disorders [17–19], and a score of < 36 (near our definition of poor MH at an MCS score of ≤35.76) has been shown to have a sensitivity of 0.62 for any depressive disorder and of 0.73 for generalised anxiety disorder. Furthermore, the probability of not having a depressive disorder at an MSC score of > 48 (which is close to our definition of good MH at an MCS score of > 48.26) is 99% with a prevalence of 3.0% [18]. Moreover, a cut-point of ≤36 seems to include individuals with severe psychological symptoms and individuals with moderate to severe disability [19, 36]. Therefore, we believe that the cut-offs for poor and good MH are reasonable for raising the GP’s awareness of their patients’ need of mental healthcare.

A limitation was that we had no information on informal or unrecorded mental healthcare, such as unrecorded psychological support by GPs, counselling outside the health services, or consultations with psychologists covered by private health insurance or self-financed consultations without referral from the GP.

We defined psychometric testing by the GP as a type of MH support. We are well aware that this is not a treatment by itself. However, we consider the use of psychometric tests in general practice as a way of taking action on MH status, and therefore as an important indicator of the mental healthcare trajectories.

We defined cessation as no recorded mental healthcare in 6 months. However, it is not very likely that one stops taking the medication on the day when a prescription is redeemed at the pharmacy (which is the date registered in the DNPR). Hence, we have categorised some participants as having ceased the medication too early in proportion to the true date of cessation. Additionally, psychotropic medication should be gradually reduced rather than stopped abruptly [37]. Thus, dose reductions that did not lead to full cessation within follow-up and unsuccessful attempts of cessation were not registered. Therefore, our estimate of individuals who had their psychotropic medication ceased is most likely an underestimation of GP action on the feedback on MH status.

Only participants with a risk profile from the health check were recommended a follow-up consultation with their GP. Therefore, cessation of mental healthcare among participants with good MH and no health risk factors was dependent on the participant’s own initiative to contact the GP or that the GP brought up the issue at the next consultation. This may have reduced the potential to cease unnecessary MH treatment.

Although both initiation and cessation of mental healthcare seemed to be time related to the MH screening, we should not draw conclusions on the causality of the observed associations in this descriptive cohort study. We obtained the MH status only on the 51% of the invited population who participated in the MH screening. Hence, we were unable to compare their mental healthcare trajectories with those of non-participants. Therefore, randomised controlled trials are needed to further explore the causal effect of MH screening on initiation and cessation of mental healthcare.

### Perspectives

Future research on the *Check Your Health* programme should take an interest in the cost of implementation versus the potential saving made by cessation of care. Another important issue to investigate in a public-based health preventive programme like the *Check Your Health* programme is the allocation of mental healthcare after participation. We are conducting a study on this issue to investigate which socio-demographic factors are associated with non-initiation of mental healthcare, despite being detected with poor MH on screening.

## Conclusion

This large-scale population-based cohort study, which was performed in a real-life setting, is unique in suggesting that MH screening followed by feedback on both positive and negative screening results to the GP contribute to both initiation and cessation of mental healthcare. The screening may be a useful tool in order to increase awareness on treatment. However, both initiation and cessation of mental healthcare can still be further targeted, and the initiative described cannot stand alone.

## Additional file

Additional file 1: **Table S1.** Socio-demographic characteristics of the SF-12 non-respondents. (DOCX 17 kb)

## Abbreviations

ACT: Anatomical therapeutic chemical
CI: Confidence interval
CVD: Cardiovascular disease
DNPR: Danish National Prescription Register
GP: General practitioner
MCS: Mental component summary
MH: Mental health
NHS: National Health Service
NHSR: National Health Service Register
NPR: National Patient Register
SF-12: 12-item Short Form Health Survey
WHO: World Health Organization

## References

[1] European Commission (2005). Green paper: improving the mental health of the population: towards a strategy on mental health for the European Union. Brussels.

[2] World Health Organization (2004). Prevention of mental disorders: effective interventions and policy options: summary report.

[3] World Health Organization (2008). The global burden of disease: 2004 update.

[4] Central Denmark Region: Psykiatriplan: Bedre behandling og længere liv til flere med psykisk sygdom på patientens præmisser - Psykiatriplan for Region Midtjylland 2017 [Psychiatry plan: Better treatment and longer life to more patients with psychiatric disease on the patient's terms - Psychiatry plan for Central Denmark Region 2017]. Viborg: Central Denmark Region; 2017. Available at: https://www.rm.dk/siteassets/sundhed/fremtidens-sundhedsvasen/psykiatriplan-2017/psykiatriplan-2017.pdf. Accessed on 30 October 2018.

[5] Mitchell AJ, Vaze A, Rao S (2009). Clinical diagnosis of depression in primary care: a meta-analysis. Lancet.

[6] Nordgaard J, Sass LA, Parnas J (2013). The psychiatric interview: validity, structure, and subjectivity. Eur Arch Psychiatry Clin Neurosci.

[7] Gidding LG, Spigt M, Winkens B, Herijgers O, Dinant G-J. PsyScan e-tool to support diagnosis and management of psychological problems in general practice: a randomised controlled trial. Br J Gen Pract. 2017. 10.3399/bjgp17X694109 PMC5737316 29255109

[8] Gilbody S, Sheldon T, House A (2008). Screening and case-finding instruments for depression: a meta-analysis. CMAJ.

[9] Eveleigh R, Muskens E, Lucassen P, Verhaak P, Spijker j, van Weel C, Voshar RO, Speckens a (2017). Too much or too little antidepressant medication: difficult to change. Two rcts. Ment Health Fam Med.

[10] Maindal HT, Støvring H, Sandbaek A (2014). Effectiveness of the population-based check your health preventive programme conducted in primary care with 4 years follow-up [the CORE trial]: study protocol for a randomised controlled trial. Trials.

[11] Kessler RC, Angermeyer M, Anthony JC, De Graaf R, Demyttenaere K, Gasquet I, De Girolamo G, Gluzman S, Gureje O, Haro JM (2007). Lifetime prevalence and age-of-onset distributions of mental disorders in the World Health Organization's world mental health survey initiative. World Psychiatry.

[12] Pedersen CB (2011). The Danish civil registration system. Scand J Public Health.

[13] Statistics Denmark (1991). IDA - an integrated data base for labour market research. Main report.

[14] International Standard Classification of Education (1997) [http://www.unesco.org/education/information/nfsunesco/doc/isced_1997.htm]. Accessed 10 Aug 2018.

[15] Ware J, Kosinski M, Keller SD (1996). A 12-item short-form health survey: construction of scales and preliminary tests of reliability and validity. Med Care.

[16] Ware JE, Kosinski M, Turner-Bowker DM, Gandek B (2002). User's manual for the SF-12v2® health survey with a supplement documenting the SF-12® health survey.

[17] Vilagut G, Forero CG, Pinto-Meza A, Haro JM, de Graaf R, Bruffaerts R, Kovess V, de Girolamo G, Matschinger H, Ferrer M (2013). The mental component of the short-form 12 health survey (SF-12) as a measure of depressive disorders in the general population: results with three alternative scoring methods. Value Health.

[18] Kiely KM, Butterworth P (2015). Validation of four measures of mental health against depression and generalized anxiety in a community based sample. Psychiatry Res.

[19] Gill SC, Butterworth P, Rodgers B, Mackinnon A (2007). Validity of the mental health component scale of the 12-item short-form health survey (MCS-12) as measure of common mental disorders in the general population. Psychiatry Res.

[20] Christensen AI, Davidsen M, Kjøller M, Juel K. Mental sundhed blandt voksne danskere: analyser baseret på sundheds- og sygelighedsundersøgelsen 2005 [mental health among adult Danes: analyses based on the Danish health and morbidity survey 2005]. Copenhagen: The Danish Health Authority. 2010;90.

[21] Christiansen T (2002). Organization and financing of the Danish health care system. Health policy.

[22] Babitsch B, Gohl D, von Lengerke T (2012). Re-revisiting Andersen's behavioral model of health services use: a systematic review of studies from 1998-2011. Psycho-social medicine.

[23] Denmark S (2015). Guidelines for transferring aggregated results from statistics Denmark’s research services.

[24] Region Midtjylland: Undersøgelse af ventetiden til psykologbehandling i Region Midtjylland 2013 [Study on waiting time to psychologist treatment in Central Region Denmark 2013]. In*.* Viborg: Region Midtjylland, Nære Sundhedstilbud.

[25] McDaid D (2010). Countering the stigmatisation and discrimination of people with mental health problems in Europe.

[26] Andrade LH, Alonso J, Mneimneh Z, Wells JE, Al-Hamzawi A, Borges G, Bromet E, Bruffaerts R, de Girolamo G, de Graaf R (2014). Barriers to mental health treatment: results from the WHO world mental health surveys. Psychol Med.

[27] Moussavi S, Chatterji S, Verdes E, Tandon A, Patel V, Ustun B (2007). Depression, chronic diseases, and decrements in health: results from the world health surveys. Lancet.

[28] Hutton C, Gunn J (2007). Do longer consultations improve the management of psychological problems in general practice? A systematic literature review. BMC Health Services Research.

[29] Clark DM (2011). Implementing NICE guidelines for the psychological treatment of depression and anxiety disorders: the IAPT experience. Int Rev Psychiatry.

[30] Ten Have M, Nuyen J, Beekman A, de Graaf R (2013). Common mental disorder severity and its association with treatment contact and treatment intensity for mental health problems. Psychol Med.

[31] The MaGPIe Research Group (2005). The effectiveness of case-finding for mental health problems in primary care. Br J Gen Pract.

[32] National Institute for Health and Clinical Excellence: Depression: the treatment and management of depression in adults (updated edition). Leicester (UK): British Psychological Society. Copyright (c) The British Psychological Society & The Royal College of Psychiatrists.; 2010. 22132433

[33] Piek E, van der Meer K, Hoogendijk WJG, Penninx BWJH, Nolen WA (2011). Most antidepressant use in primary care is justified: results of the Netherlands study of depression and anxiety. PLoS One.

[34] Verbeek-Heida PM, Mathot EF (2006). Better safe than sorry? Why patients prefer to stop using selective serotonin reuptake inhibitor (SSRI) antidepressants but are afraid to do so: results of a qualitative study. Chronic Illn.

[35] Drapeau A, Boyer R, Diallo FB (2011). Discrepancies between survey and administrative data on the use of mental health services in the general population: findings from a study conducted in Québec. BMC Public Health.

[36] Sanderson K, Andrews G (2002). Prevalence and severity of mental health-related disability and relationship to diagnosis. Psychiatr Serv.

[37] Wilson E, Lader M (2015). A review of the management of antidepressant discontinuation symptoms. Ther Adv Psychopharmacol.

[38] Kildemoes HW, Sørensen HT, Hallas J (2011). The Danish national prescription registry. Scand J Public Health.

[39] Andersen JS, Olivarius NDF, Krasnik A (2011). The Danish national health service register. Scand J Public Health.

[40] Lynge E, Sandegaard JL, Rebolj M (2011). The Danish National Patient Register. Scand J Public Health.

