# Supplementary material for: Initiation and cessation of mental healthcare after mental health screening in primary care: a prospective cohort study
Source: BMC Fam Pract. 2018 Nov 17;19:176. doi: 10.1186/s12875-018-0864-9 (PMC6240182; doi:10.1186/s12875-018-0864-9)
Supplement: Supplementary file 1 — Table S1. Socio-demographic characteristics of the SF-12 non-respondents. (DOCX 17 kb) [file 12875_2018_864_MOESM1_ESM.docx]

**Table S1. Socio-demographic characteristics of the SF-12 non-respondents**

|  | Study population | | SF-12 non-respondents | |
| --- | --- | --- | --- | --- |
|  | n | % (95% CI) | n | % (95% CI) |
|  | 5970 |  | 470 |  |
| Sex |  |  |  |  |
| Women | 3018 | 50.6 (49.3-51.8) | 267 | 56.8 (52.3-61.3) |
| Men | 2952 | 49.4 (48.2-50.7) | 203 | 43.2 (38.7-47.7) |
| Age, mean | 42.3 | (42.2-42.5) | 42.9 | (42.4-43.4) |
| Cohabitional status |  |  |  |  |
| Living alone | 1297 | 21.7 (20.7-22.8) | 135 | 28.8 (24.7-33.0) |
| Cohabiting | 4669 | 78.3 (77.2-79.3 | 333 | 71.2 (67.0-75.3) |
| Education (years) |  |  |  |  |
| 0-10 | 853 | 14.5 (13.6-15.4) | 90 | 20.3 (16.5-24.0) |
| 11-14 | 3022 | 51.2 (50.0-52.5) | 235 | 52.9 (48.3-57.6) |
| > 15 | 2023 | 34.3 (33.1-35.5) | 119 | 26.8 (22.7-30.9) |

SF-12 non-respondents: individuals who were excluded due to non-completion of the SF-12.
